# *Stenotrophomonas maltophilia* in a *Rhipicephalus linnaei* tick cell culture: detection, antibiotic resistance and multi-locus sequence typing

**DOI:** 10.1007/s10482-026-02384-w

**Published:** 2026-07-17

**Authors:** Nurul Aini Husin, Shih Keng Loong, Hai Yen Lee, Norhidayu Sahimin, Phui Chyng Yap, Jing Jing Khoo, Alistair Darby, Lesley Bell-Sakyi, Benjamin L. Makepeace, Van Lun Low

**Affiliations:** 1 Tropical Infectious Diseases Research and Education Centre (TIDREC), Higher Institution of Centre of Excellence (HICoE), Universiti Malaya, 50603 Kuala Lumpur, Malaysia; 2 Institute for Advanced Studies, Universiti Malaya, 50603 Kuala Lumpur, Malaysia; 3 Institute of Infection, Veterinary & Ecological Sciences, University of Liverpool, Liverpool, L3 5RF UK

**Keywords:** *Stenotrophomonas maltophilia*, *Rhipicephalus linnaei*, Tick cell culture, Antibiotic resistance, Multi-locus sequence typing

## Abstract

Ticks harbour diverse microbial communities, but opportunistic and environmentally associated bacteria within these systems remain poorly understood. During attempts to establish primary cell cultures from surface-sterilized embryos of the dog tick *Rhipicephalus linnaei*, we detected the presence of *Stenotrophomonas maltophilia*, a multidrug-resistant environmental bacterium and emerging opportunistic pathogen. Microscopic examination of Giemsa-stained smears showed abundant extracellular rod-shaped bacteria closely associated with degenerating tick cells, with no evidence of intracellular infection. Molecular identification based on 16S and 23S rRNA gene sequencing confirmed the bacteria as *S. maltophilia*, and antibiotic susceptibility testing revealed resistance to amoxicillin-clavulanic acid, imipenem, cefoxitin, and nitrofurantoin, but susceptibility to meropenem. Multi-locus sequence typing (MLST) identified the isolate as sequence type 948 (ST948), which is a single-locus variant of ST408 and a double-locus variant of ST144, both of which are representatives of environmental and clinical *S. maltophilia* strains from diverse geographic origins. These findings provide the first evidence of *S. maltophilia* occurring in a tick-derived cell culture system and highlight the need to consider opportunistic bacteria when interpreting tick microbiome and cell culture-based studies, particularly those involving veterinary-relevant tick species.

## Introduction

Ticks are obligate hematophagous ectoparasites and globally recognized vectors of various pathogens affecting humans, livestock, and wildlife (Jongejan & Uilenberg 2004). In addition to transmitting viruses, bacteria, and protozoa, ticks harbour diverse microbiomes that include obligate endosymbionts as well as transient or environmentally acquired bacteria. These microbes can influence tick physiology, development, and vector competence, shaping both tick biology and pathogen transmission dynamics (Bonnet et al. 2017; Duron et al. 2017). However, compared to pathogenic and symbiotic bacteria, the ecology and persistence of opportunistic or environmentally associated bacteria in ticks remain poorly understood.

*Stenotrophomonas maltophilia* is an aerobic, non-fermentative Gammaproteobacterium commonly found in soil, water, plants, and various animal hosts (Brooke 2012; Said et al. 2025). Although best known as an opportunistic human-associated bacterium, it is also frequently detected in environmental samples and is intrinsically resistant to multiple antibiotics. Its presence has been reported in arthropods, including ticks, but its ecological role within these systems is unclear (Machado-Ferreira et al. 2015; Trinachartvanit et al. 2022). Possible routes of introduction into ticks include environmental exposure, acquisition during blood feeding, or unintentional introduction during laboratory handling.

Studies over the past two decades have established tick cell cultures as powerful tools for exploring tick-associated microorganisms under controlled conditions (Bell-Sakyi et al. 2007; Cabezas-Cruz et al. 2019; Salata et al. 2021). These models, including primary tick cell cultures, allow researchers to propagate bacterial agents directly from tick tissues or homogenates, encompassing those that are otherwise difficult to isolate using conventional microbiological methods (Alberdi et al. 2012; Beliavskaia et al. 2021; Bell-Sakyi et al. 2018, 2015b; Mediannikov et al. 2012; Palomar et al. 2019).

Several important pathogens, such as *Rickettsia* spp., *Anaplasma* spp., and *Ehrlichia* spp., as well as maternally inherited endosymbionts including *Spiroplasma* spp. and apathogenic *Rickettsia* spp., have been successfully propagated using these systems (Alberdi et al. 2012; Bell-Sakyi et al. 2015a, 2000; Kurtti et al. 2015; Munderloh et al. 1996, 2003; Palomar et al. 2019; Policastro et al. 1997; Simser et al. 2002; Singu et al. 2006). However, reports of opportunistic environmental bacteria being isolated through tick cell cultures remain rare. One example is the isolation of a *Mycobacterium* species from a European tick using tick cell and organ cultures (Palomar et al. 2019), highlighting the potential of these systems to reveal unexpected microbial associations.

The primary objective of the present study was to establish primary tick cell cultures from *Rhipicephalus linnaei* as part of ongoing efforts to develop tick cell resources in Malaysia. During the establishment of these cultures, we unexpectedly isolated *S. maltophilia* from the tick tissue homogenate. Because this bacterium persisted in culture despite the presence of antibiotics, we subsequently performed molecular characterization using multi-locus sequence typing (MLST) and evaluated its antibiotic resistance profile. These analyses provide insight into the identity and potential origins of the isolate and demonstrate the utility of primary tick cell cultures as a platform for detecting incidental or opportunistic bacteria associated with ticks.

## Materials and methods

### Source of ticks

Engorged female *Rhipicephalus* sp. ticks were collected from dogs in Perak, Malaysia, with informed consent from the owner in December 2022. Following collection, the ticks were transported at room temperature to the Tick Cell Biobank Asia Outpost at the Tropical Infectious Diseases Research and Education Centre (TIDREC), Universiti Malaya, Malaysia, for further processing. Morphological identification to species level was performed using a Nikon SMZ800N stereomicroscope (Nikon Solutions Co., Ltd, Japan) and published taxonomic keys (Šlapeta et al. 2022; Tanskul & Inlao 1989).

Surface sterilization was carried out in a horizontal laminar flow cabinet prior to oviposition. Ticks were immersed in 0.1% benzalkonium chloride for 5 min, followed by 70% ethanol for 1 min, and then rinsed twice in sterile deionized water. After sterilisation, the ticks were dried on sterile filter paper and individually placed into sterile 35 mm plastic Petri dishes. The ticks were maintained in a sealed plastic box at 100% relative humidity and incubated at 28 °C. Ticks were inspected daily for oviposition and embryonic development.

### Initiation of primary tick cell cultures

Once the rectal sacs of the developing embryos were visible as white spots within the eggs, the female tick was removed. In most cases, egg batches from multiple females of the same species were pooled to generate sufficient material for a single primary culture (Table 1). Each pool of egg batches was surface-sterilized by immersion in 70% ethanol for 1 min, followed by two rinses in sterile Hanks’ balanced salt solution. The eggs were gently crushed with the flattened end of a glass rod in complete culture medium to release the embryonic tissues. The resulting suspension was transferred to a flat-sided culture tube (Nunc, Thermo-Fisher, UK) and incubated horizontally at 28 °C in a dry incubator with ambient air (Bell-Sakyi et al. 2022; Palomar et al. 2019). Medium was changed weekly by removal and replacement of part of the volume, being careful not to remove any of the tick tissues. All manipulations were performed under aseptic conditions in a Class II microbiological safety cabinet using sterilized instruments and filtered reagents to minimize the risk of contamination.

**Table 1:** Egg batches and media used for initiation of *Rhipicephalus linnaei* primary cultures

Culture tube ID	No. of egg batches	Medium
T1	1	L-15
T2	6	L-15
T3	6	L-15
T4	5	L-15B
T5	3	L-15B
T6	3	L-15B
T7	2	L-15/MEM
T8	3	L-15/MEM

Three complete culture media were used, each supplemented with 2 mM L-glutamine, 100 U/mL penicillin, and 100 µg/mL streptomycin, and freshly prepared weekly. Medium components were obtained from Invitrogen (Thermo Fisher, UK) or Sigma (Sigma Aldrich, UK), unless otherwise indicated. Complete L-15 comprised L-15 (Leibovitz) medium supplemented with 10% tryptose phosphate broth (TPB) and 20% foetal bovine serum (FBS). Complete L-15/MEM combined equal parts of L-15 (Leibovitz) and Minimal Essential Medium with Hanks’ salts, also supplemented with 10% TPB and 20% FBS. Complete L-15B comprised basal L-15B medium (Munderloh & Kurtti 1989) supplemented with 10% TPB, 5% FBS, and 0.1% bovine lipoprotein concentrate (MP Biomedicals, Thermo Fisher, UK).

### Routine maintenance and microscopic examination

Primary cultures were monitored weekly using a BMI-100 inverted microscope (Biobase, China) prior to changing the medium. Observations included cell proliferation, tissue attachment, and overall culture condition. During each medium change, between half and three-quarters of the volume was replaced with fresh medium.

To visualise bacteria, 50 µL of cell suspension was centrifuged onto glass microscope slides for 5 min at 1000 rpm using an Epredia™ Cytospin™ 4 cytocentrifuge (Thermo Fisher Scientific, UK). The smears were air-dried, fixed in methanol for 3 min, stained with Giemsa (Merck, Germany), and rinsed three times with buffered deionised water (pH 7.2). Cytocentrifuge smears were examined at 1000 × magnification under a compound microscope (GX Microscopes, UK) and photographed using a GXCAM digital camera with GXCapture software.

### Cryopreservation of infected cultures

Tick cell cultures containing bacteria (detected by microscopy and/or PCR) were resuspended gently, kept on ice, and mixed with dimethyl sulphoxide (DMSO) to a final concentration of 10%. The suspension was then immediately aliquoted into pre-chilled, labelled cryovials, which were rapidly frozen on dry ice and subsequently stored in the vapour phase of a liquid nitrogen tank.

### DNA extraction and PCR for bacterial detection and identification

DNA was extracted from 200 µL of cell suspension using the DNeasy Blood and Tissue Kit (Qiagen, Germany) following the manufacturer’s instructions for cultured cells. For extraction, both adherent and floating cells were collected from each culture by gentle resuspension and centrifugation at 10,000 × *g* for 5 min to obtain a uniform cell pellet. The pellet was resuspended in phosphate-buffered saline to a final volume of 200 µL prior to lysis. The extracted DNA was subsequently used for PCR-based bacterial screening and species confirmation.

Initial screening was conducted using a universal 16S rRNA primer set targeting a 528-bp fragment: Eubac27F (5′-AGAGTTTGATCCTGGCTCAG-3′) and 518R (5′-GWATTACCGCGGCTGCT GG-3′) (Benson et al. 2004). The PCR cycling conditions included an initial denaturation at 94 °C for 5 min, followed by 35 cycles of 94 °C for 30 s, 50 °C for 30 s, and 72 °C for 30 s, and a final extension at 72 °C for 7 min.

Following 16S rRNA amplification using the universal primer set, bacterial identification was confirmed using species-specific primers targeting *S. maltophilia*. Specifically, a fragment of the 23S ribosomal RNA (23S rRNA) gene was amplified using the primer pairs: SM1 (5'-CAGCCTGCGAAAAGTA-3’) and SM4 (5'-TTAAGCTTGCCACGAACAG-3’), and 23SRNA3 (5'-GAATATTGACCTGCTTCCC-3’) and SM23RNA3 (5'-GGTCAAGCGAATAAGCGC-3’) (Whitby et al. 2000). All PCR reactions were conducted in 25 μL volumes using 2 × MyTaq® Red Mix (Bioline, UK), with 0.4 μM of each primer and 2 μL of template DNA. The PCR cycling conditions were an initial denaturation at 95℃ for 5 min, 30 cycles of denaturation at 95℃ for 10 s, annealing at 58℃ for 10 s, and extension at 72℃ for 1 min, followed by the final extension at 72℃ for 10 min and final hold at 4℃.

Validated primers were used for amplification, and each PCR run included standard positive and negative controls (nuclease-free water) to ensure both assay sensitivity and specificity. For pan-bacterial PCRs, DNA extracted from tick cells infected with *Rickettsia raoultii* (strain Białystok1), isolated from a Polish *Dermacentor reticulatus* tick (Palomar et al. 2019), was used as a positive control.

### Isolation and identification of bacteria

Following microscopic observation of bacterial growth in the primary tick cell culture, and prior to cryopreservation, 100 µL of the infected culture suspension was plated onto Mueller Hinton agar (MHA) (Himedia, India) and incubated at 37 °C for three days. Subculture was performed until pure bacterial colonies were obtained. A single colony was then picked and subjected to DNA extraction using the boiling method as follows: the colony was resuspended in 200 µL of sterile distilled water, heated at 95 °C for 10 min, and immediately transferred to ice for 5 min. The suspension was centrifuged, and the supernatant containing crude genomic DNA was collected. This DNA extract was used directly as the final template for downstream PCR analysis, without further purification.

To identify the bacterial isolate, a pan-bacterial PCR targeting the nearly full-length 16S rRNA gene was performed using universal bacterial primers 27F (5′-AGAGTTTGATCMTGGCTCAG-3′) and 1492R (5′-TACGG YTACCTTGTTACGACTT-3′) (Heuer et al. 1997). The PCR conditions were: initial denaturation at 95 °C for 2 min; 35 cycles of 95 °C for 30 s, 49 °C for 30 s, and 72 °C for 1 min, followed by final extension at 72 °C for 10 min and hold at 4 °C.

### DNA sequencing and analysis

The amplified PCR products were visualized on a 1.0% (w/v) agarose gel stained with SYBR® Safe nucleic acid stain (Invitrogen Life Technologies, California). Amplicons were sent for DNA sequencing to a third-party service provider (Apical Scientific Sdn Bhd, Malaysia). Pair-end sequences were assembled into consensus sequences using BioEdit software version 7.7.1 (Hall 1999). These sequences were then compared against the National Center for Biotechnology Information (NCBI) GenBank database via Basic Local Alignment Search Tool (BLAST) for species identification (http://www.ncbi.nml.ni h.gov/BLAST/).

### Antibiotic susceptibility test

The Kirby-Bauer disk diffusion method was performed according to CLSI guidelines (Clinical and Laboratory Standards Institute, 2022). In brief, a single colony of each bacterial strain, previously isolated and purified, was transferred to Mueller Hinton broth (Himedia, India) and incubated at 25 °C overnight to reflect conditions compatible with tick cell culture. The overnight culture was centrifuged, and the cell pellet was washed twice. The bacterial culture was adjusted to a 0.5 McFarland standard (approximately 10^8^ CFU/mL) at an optical density of 600 nm. The culture was then diluted to 10^6^ CFU/mL and spread onto MHA.

Antibiotic susceptibility was tested using four different antibiotic rings (Combi 505, Combi 506, Combi 516, and Universal-1) (Himedia, India), consisting of the antibiotics listed in Table 3, with incubation at 37 °C overnight. Among the antibiotics tested, trimethoprim-sulfamethoxazole, levofloxacin and minocycline are those agents for which CLSI has established interpretive minimum inhibitory concentration (MIC) breakpoints for *S. maltophilia*. All assays were performed in duplicate, and consistent, reproducible results were obtained.

### Multi-locus sequence typing and construction of minimum spanning tree

MLST was performed on the bacterial isolates according to the *S. maltophilia* MLST scheme (Kaiser et al. 2009). Seven loci (*atpD*, *gapA*, *guaA*, *mutM*, *nuoD*, *ppsA* and *recA*) were amplified, sequenced, and analysed according to protocols available at the PubMLST database (https://pubmlst.org/organisms/stenotrophomonas-maltophilia) (Jolley et al. 2018). PHYLOViZ Online was subsequently used to generate a minimum spanning tree using the MLST results in order to display the genetic relatedness between the bacterial isolates obtained in the current study and those found in the PubMLST database (Chua et al. 2024).

## Results

### Establishment of *R. linnaei *primary cell cultures and detection of *S. maltophilia*

Eight primary cultures were initiated from single or pooled egg batches laid by 29 engorged *R. linnaei* females collected from dogs in Perak, Malaysia (Table 1). Over a six-month observation period, most cultures exhibited limited cell growth. However, one culture (tube T4) developed marked cytopathic changes, including progressive cell shrinkage, disintegration and eventual loss of intact cells.

**Fig. 1:**
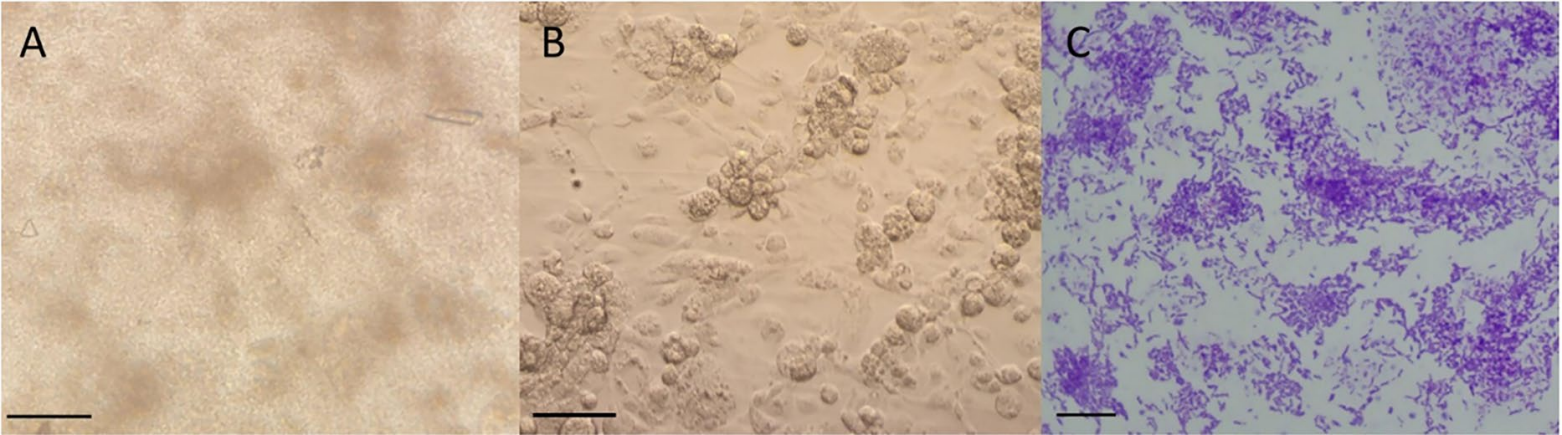
Appearance of *Stenotrophomonas maltophilia* in *Rhipicephalus linnaei* primary tick cell culture. (**A**) Phase-contrast image (scale bar = 100 µm) of infected culture T4 six months post-initiation showing cytopathic effects (CPE), including cell shrinkage, clumping, and detachment. (**B**) Phase-contrast image (scale bar = 100 µm) of an uninfected *R. linnaei* primary culture (T2, 14 months post-initiation) displaying normal morphology with intact cell membranes. (**C**) Giemsa-stained cytocentrifuge smear (scale bar = 10 µm) prepared from the infected T4 culture, showing abundant extracellular rod-shaped bacteria consistent with *S. maltophilia*

Phase contrast microscopy revealed alterations in cell morphology, characterized by cell shrinkage (Fig. 1A), and a loss of the typical spindle-shaped and rounded cell appearance seen in healthy tick cell cultures (Fig. 1B). A Giemsa-stained cytocentrifuge smear confirmed a heavy bacterial presence and an absence of intact tick cells (Fig. 1C). No bacterial contamination was detected in the remaining primary cultures, indicating that the bacterial growth in T4 was a singular event.

Although the culture medium contained penicillin (100 U/mL) and streptomycin (100 µg/mL), antibiotics commonly used during primary culture establishment, the bacteria continued to proliferate. This observation is consistent with the well-documented intrinsic resistance of *S. maltophilia* to β-lactams and aminoglycosides, and may have contributed to the deterioration of the T4 culture.

### Molecular identification of the bacterial isolate

Molecular screening using the universal 16S rRNA PCR detected bacterial DNA in the T4 culture. Sequencing of the 528 bp amplicon revealed 100% identity with *Stenotrophomonas* sp. LQX-11 (Accession no. DQ256392). To further confirm the identification, two PCR assays targeting the *S. maltophilia* 23S rRNA gene were performed on the T4 culture, with the resultant sequences demonstrating 100% (531 bp amplicon) and 99.92% (1,381 bp amplicon) identity to *S. maltophilia* strain 142 (Accession no. CP098483). The obtained DNA sequences were deposited in GenBank under the following accession numbers: PV849137, PV849141 and PV849142 (Table 2).

**Table 2:** Sequence data and BLAST results for *Stenotrophomonas* isolates from this study with closest GenBank matches, including identity, query cover, and geographic origin

Gene target (Sample)	Amplicon size (bp)	Species identity	Study GenBank accession no	Closest GenBank match	Identity (%)	Query cover (%)	Geographic origin of match
Universal 16S rRNA (T4 culture)	528	*Stenotrophomonas* sp.	PV849137	DQ256392	100	100	China
*S. maltophilia* 23S rRNA (T4 culture)	531	*S. maltophilia*	PV849141	CP098483	100	100	China
*S. maltophilia* 23S rRNA (T4 culture)	1,381	*S. maltophilia*	PV849142	CP098483	99.92	100	China
16S rRNA, nearly full-length (L15a)	1,465	*S. maltophilia*	PV849138	OP896973	98	98.57	China
16S rRNA, nearly full-length (L15b)	1,465	*S. maltophilia*	PV849139	ON698109	99	98.51	China

A 100 µL inoculum from the T4 culture that was transferred to an MHA plate and incubated at 37 °C for three days grew two different colonies with similar phenotypic characteristics. There was a slight difference in colour tone between the two colonies, hence one colony was designated L15a (white) and the other L15b (pale yellow). Sequencing of the nearly full-length 16S rRNA gene identified both isolates (L15a and L15b) as *S. maltophilia*. The obtained DNA sequences were deposited in GenBank under the following accession numbers: PV849138 and PV849139 (Table 2).

### MLST analysis of *S. maltophilia* isolates

**Fig. 2:**
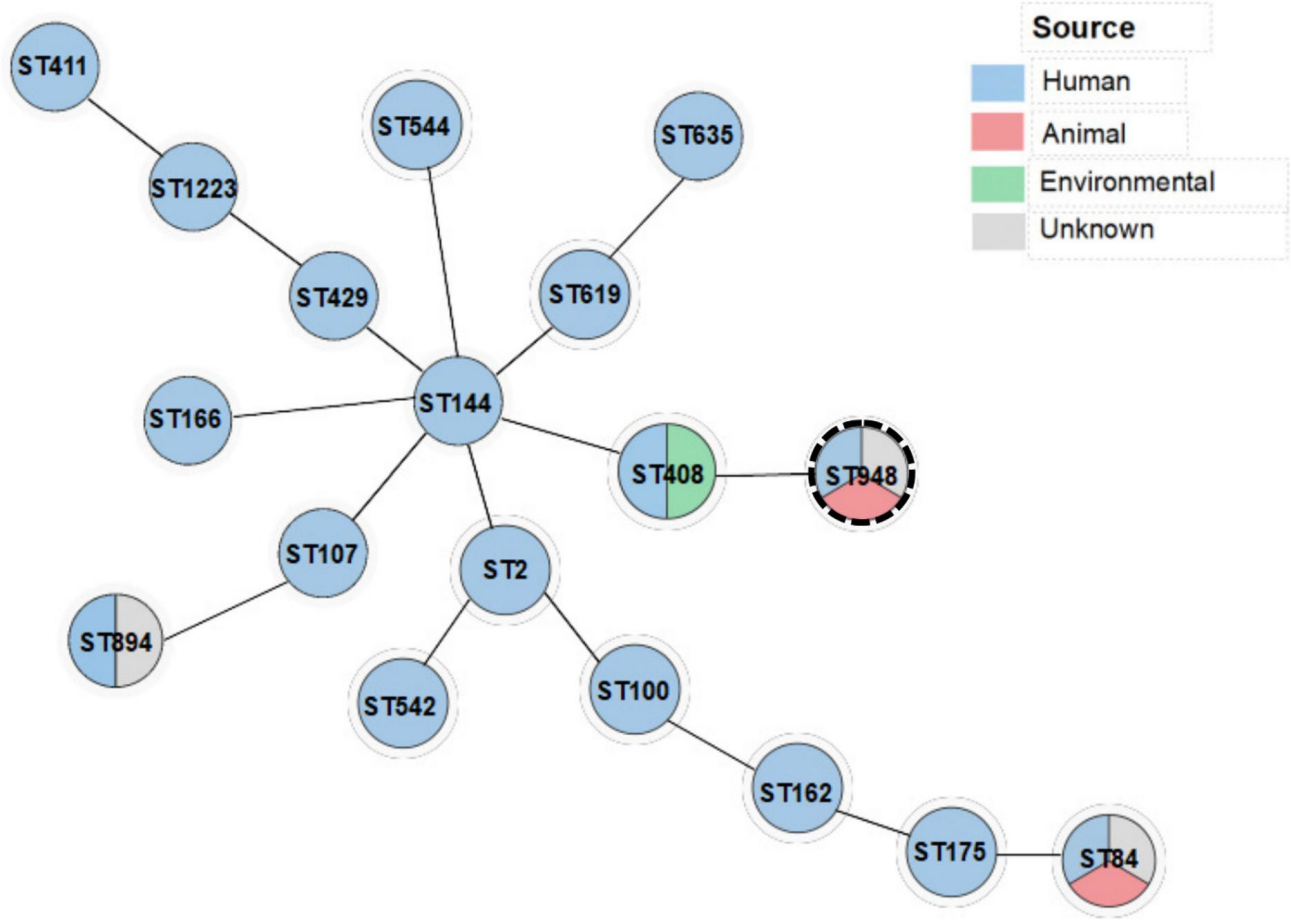
Minimum spanning tree demonstrates the genetic relatedness between *Stenotrophomonas maltophilia* ST948 and other *S. maltophilia* MLST profiles from the PubMLST database. Each node represents a unique sequence type, and colours indicate the source of each isolate. The ST948 profile, including the isolates from this study, is highlighted with a black dashed border. Connecting lines represent allelic differences between sequence types; shorter connections indicate closer genetic relationships. Branch lengths are schematic and not to scale

MLST was conducted for both L15a and L15b isolates based on seven housekeeping genes. Identical allelic profiles were observed for both isolates: *atpD* (allele 1), *gapA* (allele 4), *guaA* (allele 336), *mutM* (allele 51), *nuoD* (allele 25), *ppsA* (allele 38), and *recA* (allele 1). This allelic combination corresponds to sequence type 948 (ST948). A minimum spanning tree based on MLST data showed that ST948 is closely related to ST408 (single-locus variant) and ST144 (double-locus variant) (Fig. 2).

### Antibiotic susceptibility profile

Antibiotic susceptibility testing was performed for the two *S. maltophilia* isolates, L15a and L15b. Despite slight genetic differences, both isolates exhibited identical antibiotic susceptibility profiles. Of the 20 antibiotics tested, the isolates were resistant to imipenem (IPM), cefoxitin (CX), amoxiclav (AMC), and nitrofurantoin (NIT), while being susceptible to meropenem (MRP) and the remaining antibiotics tested (Table 3).

**Table 3:** Antibiotic susceptibility profile of *Stenotrophomonas maltophilia* isolates L15a and L15b

Antibiotic group	Antibiotic ^[1]^	Concentration of antibiotic (μg)	Susceptibility ^[2]^	
			L15a	L15b
Carbapenem	IPM	10	R	R
	MRP	10	S	S
^†^Fluoroquinolone	CIP	5	S	S
	GAT	5	S	S
	OF	5	S	S
Macrolides	AZM	15	S	S
Oxazolidinone	LZ	30	S	S
Quinolone	MO	5	S	S
	SPX	5	S	S
Cephamycins	CX	30	R	R
Aminoglycosides	AK	30	S	S
	GEN	10	S	S
^†^Tetracyclines	DO	30	S	S
	TE	30	S	S
Cephalosporin	CPZ	75	S	S
	CPD	30	S	S
β-lactam combination agents	AMC	30	R	R
Dihydrofolate reductase inhibitors	COT	23.75/1.25	S	S
Phenicols	C	30	S	S
Nitrofurantoins	NIT	300	R	R

^[1]^ AK-Amikacin; AMC-Amoxiclav (amoxicillin/clavulanic acid); AZM-Azithromycin; C-Chloramphenicol; CIP-Ciprofloxacin; COT-Co-trimoxazole (Sulpha/Trimethoprim); CPD- Cefpodoxime; CPZ-Cefoperazone; CX-Cefoxitin; DO-Doxycycline hydrochloride; GAT-Gatifloxacin; GEN-Gentamicin; IPM-Imipenem; LZ-Linezolid; MO-Moxifloxacin; MRP-Meropenem; NIT-Nitrofurantoin; OF- Ofloxacin; SPX-Sparfloxacin; TE-Tetracycline

^[2]^ R-Resistant; S-Susceptible

^†^ Antibiotic group stated in the Clinical and Laboratory Standards Institute (CLSI) for *S. maltophilia*

## Discussion

This study reports the detection and isolation of *S. maltophilia* from one out of eight primary *R. linnaei* embryo-derived cell cultures. All primary cultures were established under standard aseptic conditions with routine surface sterilization, and the bacterium was only recovered from the single initially positive culture. Although contamination cannot be fully excluded, the absence of *S. maltophilia* from seven other primary cultures generated from similar starting material using the same stringent surface-sterilization protocol is consistent with, but does not prove, a tick-derived origin of the bacterium. The plausibility of a tick-associated origin accords with a previous report of *S. maltophilia* from tick eggs (Machado-Ferreira et al. 2015), although this does not constitute direct evidence in the present study. Hence, as no PCR screening of intact eggs, environmental controls, or replicate culture batches was performed, the source of the bacterium cannot be determined conclusively. Accordingly, the findings should be interpreted as an incidental detection of *S. maltophilia* in a tick-derived culture system rather than definitive evidence of a natural association with *R. linnaei*.

Furthermore, because *S. maltophilia* was recovered from only one of eight primary cultures established in this study, the findings should be interpreted as a single observational event. Although two colony morphotypes were recovered, MLST analysis demonstrated that both isolates belonged to the same sequence type (ST948), indicating that they do not represent independent strains. Besides, the finding was not replicated in additional culture batches, and therefore its reproducibility remains to be established. Further studies involving larger numbers of cultures, biological replicates, and independent isolates will be necessary to assess the prevalence, reproducibility, and biological significance of *S. maltophilia* in tick-derived culture systems.

Microscopic examination of Giemsa-stained smears revealed abundant extracellular, rod-shaped bacteria adhering to and surrounding degenerating tick cells, with no evidence of intracellular infection. These observations indicate that *S. maltophilia* multiplied extracellularly under the tested culture conditions. However, the present study did not include quantitative measurements of bacterial load, growth kinetics, or proliferation rates. Consequently, the inference of bacterial proliferation is based on qualitative microscopic observations and successful recovery of viable isolates from the affected culture. Future studies incorporating quantitative approaches such as qPCR, CFU enumeration, or longitudinal growth analyses would provide a more detailed understanding of bacterial growth dynamics in tick-derived culture systems.

Previous studies have reported bacterial colonization of tick eggs and internal structures, including with *S. maltophilia* in *Amblyomma cajennense* (Machado-Ferreira et al. 2015), and its detection within tick-associated microbial communities in *Amblyomma americanum* (Heise et al. 2014) and *Ixodes ricinus* (Okła et al. 2012). The recovery of *S. maltophilia* from surface-sterilized *R. linnaei* eggs is consistent with these observations, suggesting that diverse bacteria may occur naturally within tick developmental stages. However, the presence of *S. maltophilia* in eggs does not demonstrate vertical transmission. Potential routes include transovarial passage through female reproductive tissues or low-level contamination during oviposition. Transmission assays incorporating multiple egg batches and controlled contamination-tracking should be used in future work to determine whether *S. maltophilia* can be maternally transmitted.

Under in vitro conditions, *S. maltophilia* demonstrated the ability to survive and multiply in a primary tick cell culture despite the presence of penicillin and streptomycin. The presence of abundant extracellular bacteria was associated with progressive deterioration of the culture, including cytopathic changes and the loss of intact tick cells. However, because no experimental studies were performed to directly assess bacterial pathogenicity or quantify bacterial burden over time, a causal relationship between bacterial proliferation and the observed culture deterioration cannot be established. The observed association is consistent with the known ability of *S. maltophilia* to produce extracellular proteases, lipases, toxins, and biofilms (Brooke 2012). The ability of the bacterium to persist despite antibiotic supplementation is consistent with the intrinsic resistance characteristics of *S. maltophilia*.

Molecular identification based on 16S and 23S rRNA gene sequencing showed > 99% identity to reference *S. maltophilia* strains, and MLST typing revealed that both isolates belonged to sequence type ST948. Species identification was supported by concordant results from 16S rRNA gene sequencing, species-specific 23S rRNA PCR assays, and MLST analysis, providing robust evidence for assignment of the isolates to *S. maltophilia*. The ST948 isolate has been reported from both environmental and clinical settings. Comparative analysis using the PubMLST database showed that ST948 is closely related to ST408 (a single-locus variant isolated from environmental and clinical sources in Brazil and China) and ST144 (a double-locus variant from human blood in Brazil) (Rizek et al. 2018; Yinsai et al. 2023). These relationships indicate that ST948 belongs to a lineage that includes sequence types previously reported from both environmental and clinical sources. However, MLST-based affinity alone does not establish ecological connectivity, transmission pathways, or epidemiological associations among isolates from different hosts or environments. Further genomic and epidemiological investigations would be required to clarify these relationships.

Antibiotic susceptibility testing showed a multidrug-resistant phenotype typical of *S. maltophilia*, including resistance to key β-lactams such as imipenem and amoxicillin–clavulanate. These results align with the known activity of L1/L2 β-lactamases (Brooke 2012; Chang et al. 2015), whereas the isolate remained susceptible to meropenem, a pattern reported in some environmental strains (Adegoke & Okoh 2014). These observations provide baseline information on the antimicrobial profile of a tick-associated isolate and suggest that antimicrobial responses may vary among isolates from clinical, environmental, and arthropod-associated sources.

As the study focused on the exploratory isolation and characterization of a tick-associated *S. maltophilia* isolate, the antimicrobial susceptibility testing was performed to establish a preliminary phenotypic resistance profile as part of environmental surveillance. The antimicrobial panel was selected to provide broad resistance profiling as baseline data for future surveillance taking into consideration the utilization of compounds relevant to environmental exposure, veterinary use, and agents of clinical importance in human medicine. The absence of reference quality control strains and MIC determination is acknowledged as a limitation of this exploratory study; future work should incorporate standardized control strains and expanded susceptibility panels to enable more rigorous comparative interpretation.

Although *S. maltophilia* is widely regarded as a low-virulence, opportunistic pathogen in humans, particularly in immunocompromised individuals, it is increasingly found in environmental reservoirs and arthropod hosts. Recent studies have reported its presence in insects and mites, including *Tenebrio molitor* (Ye et al. 2024), *Bactrocera oleae* (Blow et al. 2016), *Phytoseiulus persimilis* (Yan et al. 2025), and *Dendroctonus rhizophagus* (Morales-Jiménez et al. 2012), where it may play roles in nutrient metabolism, defence, or microbiome stability. In the predatory mite *Neoseiulus californicus*, *S. maltophilia* was linked to improved breeding performance (Andrianov et al. 2024), suggesting that interactions between this bacterium and arthropods may vary across host species and ecological contexts.

The *R. linnaei* ticks used for culture in this study originated from dogs, a primary host species across tropical regions. Previous studies have reported the detection of *S. maltophilia* in an *R. sanguineus s.l.* tick (Trinachartvanit et al. 2022) and its isolation from canine dermatological infections (Rajeev et al. 2025). While these observations indicate that *S. maltophilia* can occur in both tick- and canine-associated contexts, the present study does not establish any epidemiological or biological relationship between the tick-derived isolate and canine disease. Whether *S. maltophilia* influences tick physiology or interacts with other members of the tick-associated microbiota remains unknown and warrants further investigation.

## Conclusions

This study documents the presence and extracellular proliferation of *S. maltophilia* in a primary *R. linnaei* embryo-derived cell culture. The bacterium was identified through microscopic observation and confirmed by amplification and sequencing of the 16S and 23S rRNA genes. Its proliferation in primary cell culture was associated with cytopathic changes and the loss of intact tick cells, indicating that the bacterium was able to persist within the culture system under the conditions tested; although the present study does not establish a causal relationship between bacterial proliferation and the observed culture deterioration. Genetic typing assigned two isolates derived from the tick cell culture to ST948, a sequence type related to strains previously reported from both environmental and clinical settings. However, the present study does not establish epidemiological, pathogenic, or transmission links between the tick-derived isolate and human or veterinary disease. Antibiotic susceptibility profiling revealed resistance to key β-lactams, including imipenem and amoxicillin-clavulanate, consistent with the intrinsic resistance mechanisms of *S. maltophilia*. The findings expand current knowledge of the occurrence of *S. maltophilia* in tick-derived culture systems and provide baseline phenotypic and genotypic data for a tick-associated isolate, but additional observations are needed to determine whether *S. maltophilia* forms a natural association with *R. linnaei* or persists within ticks under natural conditions. Accordingly, investigations involving direct screening of ticks and tick eggs, together with ecological and transmission studies, are required to clarify the biological relevance of this finding.

## Data Availability

The DNA sequences generated and analyzed during the current study have been deposited in the GenBank repository under accession numbers PV849137, PV849138, PV849139, PV849141, and PV849142.
